# IgG4‐related disease in the differential diagnosis of lung nodules

**DOI:** 10.1002/rcr2.550

**Published:** 2020-03-10

**Authors:** Hiroki Ishikawa, Hironori Uruga, Takeshi Fujii, Atsuko Kurosaki, Nasa Morokawa, Hisashi Takaya

**Affiliations:** ^1^ Department of Respiratory Medicine Toranomon Hospital Kajigaya Kawasaki Japan; ^2^ Department of Respiratory Medicine, Respiratory Center Toranomon Hospital Tokyo Japan; ^3^ Okinaka Memorial Institute for Medical Research Tokyo Japan; ^4^ Department of Pathology Toranomon Hospital Kajigaya Kawasaki Japan; ^5^ Department of Diagnostic Radiology Japan Anti‐Tuberculosis Association, Fukujuji Hospital Tokyo Japan

**Keywords:** IgG4‐related lung disease, IgG4‐related pleuritis, lung cancer, pleural dissemination

## Abstract

IgG4‐related disease is an evolving entity of immune‐mediated origin. We report a case of IgG4‐related disease mimicking lung cancer with pleural dissemination. A 76‐year‐old male non‐smoker was admitted to our hospital because of chest X‐ray abnormality. Chest computed tomography scan showed a lung nodule measuring 26 × 14 mm with tiny nodules on the adjacent pleural surface. Wedge resection by video‐assisted thoracoscopic surgery (VATS) was performed to aid diagnosis. Pathological findings of the nodule consisted of lymphoid follicular hyperplasia with lymphoplasmacytic infiltrate, fibrosis, and obstructive vasculitis. Focal and scattered thickening of the pleura with lymphoplasmacytic infiltrate was also observed. The IgG4/IgG ratio in the most prominent area exceeded 80%. Thus, we made a diagnosis of IgG4‐related lung and pleural disease. To our knowledge, there has been no report of IgG4‐related lung disease mimicking lung cancer with pleural dissemination.

## Graphical abstract

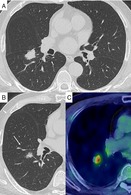

## Introduction

Research on IgG4‐related diseases began when increased IgG4‐positive plasma cells in lung nodules and autoimmune pancreatitis were reported 1, 2. Later studies revealed that IgG4‐related diseases can cause various organ dysfunction including lung and pleural disease, Mikulicz syndrome, sclerosing cholangitis, ocular and orbital inflammatory disease, retroperitoneal fibrosis, thyroiditis, and tubulointerstitial nephritis 3, 4. Because radiological and pathological findings of IgG4‐related lung disease varied, the differential diagnoses are also diverse and include lung cancer, inflammatory myofibroblastic tumour, sarcoidosis, granulomatosis with polyangiitis, Castleman disease, lymphomatoid granulomatosis, and interstitial pneumonia 4. Here, we report a case of IgG4‐related lung and pleural disease, which we successfully diagnosed, and excluded lung cancer with pleural dissemination.

## Case Report

**Figure 1:**
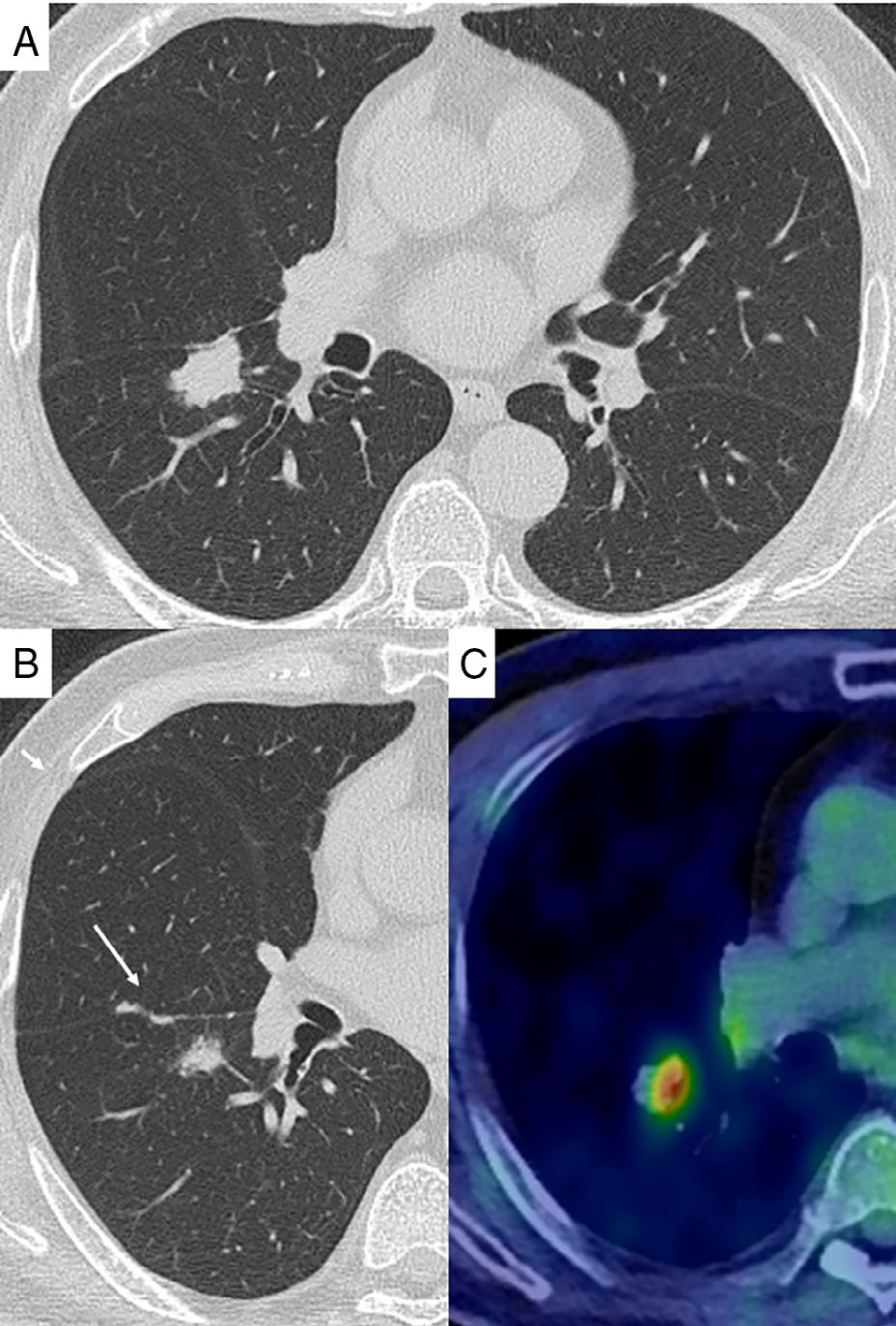
(A, B) Chest computed tomography (CT) scan showed a lung nodule measuring 26 × 14 mm with multiple tiny nodules on the pleural surface (arrow). (C) ^18^F‐fluorodeoxyglucose‐positron emission tomography scan showed a maximum standardized uptake value of 4.69 within the nodule.

**Figure 2:**
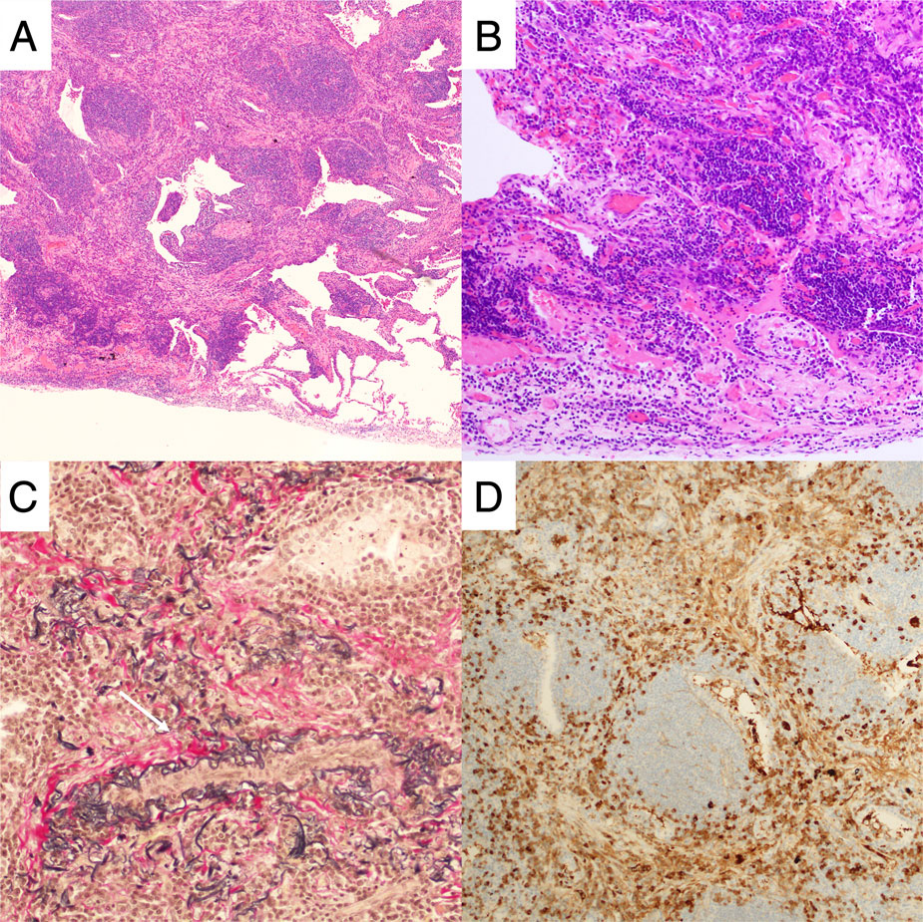
Histology of video‐assisted thoracoscopic surgery specimens included lymphoid follicular hyperplasia with lymphoplasmacytic infiltrate, fibrosis (A), thickened pleura (B), and obstructive vasculitis (C). There were prominent IgG4‐positive plasma cells and lymphocytes (D).

A 76‐year‐old man was admitted to our hospital because of chest X‐ray abnormality for a mass check‐up of lung cancer without any symptoms. He was a non‐smoker and was on medications for hypertension and type 2 diabetes (atorvastatin, combination drug of sitagliptin and ipragliflozin, candesartan, and glimepiride). Blood test revealed elevated serum IgG4 level of 266 mg/dL (normal values were less than 118 mg/dL); all other assayed tumour markers were normal. Chest computed tomography (CT) scan showed a lung nodule measuring 26 × 14 mm with multiple tiny nodules on the adjacent pleural surface (Fig. 1A, B). There was no mediastinal lymphadenopathy. ^18^F‐fluorodeoxyglucose‐positron emission tomography (^18^F‐FDG PET) scan showed a maximum standardized uptake value of 4.69 within the nodule and 3.67 within right hilar lymph node (Fig. 1C). Transbronchial lung biopsy specimens obtained via fibreoptic bronchoscopy demonstrated an infiltration of IgG4‐positive prominent plasma cell and small lymphocytes, with no evidence of malignancy. We had a high index of suspicion for IgG4‐related lung disease, but noted the need to definitively rule out lung cancer with pleural dissemination. Thus, wedge resection by video‐assisted thoracoscopic surgery (VATS) was performed. Histopathological findings of VATS specimens included lymphoid follicular hyperplasia with lymphoplasmacytic infiltrate, fibrosis, and obstructive vasculitis (Fig. 2). The IgG4/IgG ratio in the most prominent area exceeded 80%. The pleura was thickened with lymphoplasmacytic infiltrate. We thus made a diagnosis of IgG4‐related lung disease with pleuritis. There were no findings suggestive of IgG4 disease in other organs such as the pancreas and salivary glands on CT and ^18^F‐FDG PET scan. The patient came to our hospital for six months for regular follow‐up visits and was managed without steroid therapy; the subsequent clinical course was unremarkable and the patient remained well.

## Discussion

We report a case of focal and scattered pleuritis with IgG4‐positive lymphoplasmacytic infiltrate with radiological features of potential lung cancer. To our knowledge, there has been no report of IgG4‐related lung disease mimicking lung cancer with pleural dissemination.

Inoue et al. classified patterns of CT findings in patients with IgG4‐related lung disease into four categories: solid nodular type, round‐shaped ground‐glass opacity (GGO) type, alveolar interstitial type, and bronchovascular type 5. The solid nodular type is the second most common pattern, accounting for 30.8%. Lung cancer was reported to be the most important differential diagnosis for the solid nodular type and the alveolar interstitial type 5. Our case was the solid nodular type. This was the primary feature that highlighted the need to exclude lung cancer.

In our patient, pleuritis with lymphoplasmacytic infiltrate mimicked pleural dissemination of lung cancer. Zen et al. reported IgG4‐related pleuritis found in five of 21 (23.8%) patients with IgG4‐related lung disease, with sclerosing inflammation seen on pathological examination 6. There are some case reports of IgG4‐related pleuritis, but many of the patients presented with pleural effusion 7. The pleural lesions in our case were focal and scattered, and this created unevenness mimicking pleural dissemination of lung cancer. This was the second feature for differentiation.

Whilst it is crucial to exclude lung cancer in such cases, it is important to highlight that lung cancer can coexist or even complicate IgG4 disease. Fujimoto et al. examined 294 non‐small cell lung cancer surgical specimens and found IgG4‐positive plasma cell infiltrate in 35 of 294 (11.9%) specimens 8. According to Asano et al., 34 malignancies were found in patients with IgG4‐related disease over approximately six years of follow‐up, with the most common malignancy being lung cancer 9. Elevated serum IgG4 level can help in the diagnosis of IgG4 disease, although they can also be found in patients with other conditions including repeated infections, autoimmune diseases, cystic fibrosis, and cancer 10.

We report a case of IgG4‐related lung disease and pleuritis mimicking lung cancer where focal and scattered IgG4‐related pleuritis mimicked pleural dissemination of lung cancer. If we see patients with a lung nodule and elevated IgG4 level, surgical biopsy is recommended.

### Disclosure Statement

Appropriate written informed consent was obtained for publication of this case report and accompanying images.
